# Particle-Bound PAHs and Elements in a Highly Industrialized City in Southern Italy: PM_2.5_ Chemical Characterization and Source Apportionment after the Implementation of Governmental Measures for Air Pollution Mitigation and Control

**DOI:** 10.3390/ijerph17134843

**Published:** 2020-07-05

**Authors:** Jolanda Palmisani, Alessia Di Gilio, Silvana Angela Franchini, Pietro Cotugno, Daniela Valeria Miniero, Paolo D’Ambruoso, Gianluigi de Gennaro

**Affiliations:** 1 Department of Biology, University of Bari Aldo Moro, via Orabona 4, 70126 Bari, Italy; safranchini14@gmail.com (S.A.F.); pietro.cotugno@uniba.it (P.C.); danielavaleria.miniero@uniba.it (D.V.M.); gianluigi.degennaro@uniba.it (G.d.G.); 2 Regional Agency for Environmental Prevention and Protection (ARPA Puglia), Corso Trieste 27, 70126 Bari, Italy; paolo.dambruoso@libero.it

**Keywords:** PAHs, elements, particulate matter, steel plant, chemical characterization, diagnostic ratios, source apportionment, positive matrix factorization, polar plots

## Abstract

The present study was aimed at determining airborne concentrations of PAHs, Nitro-/Oxy-PAHs and elements in industrial and urban areas of Taranto, a site of environmental risk in Southern Italy, after the issue of strategic measures for air pollution mitigation and control by the Italian Environment Ministry in 2012. A PM_2.5_ sampling campaign was carried out from 9 to 28 December 2014 at eight receptor sites, two placed in the urban settlement and five included in the high spatial resolution fence monitoring network of the biggest European steel plant. The integration of collected data with meteorological parameters and source apportionment analysis by Positive Matrix Factorization and bivariate polar plots allowed to discriminate among emission sources and estimate their contributions. Evidence on the effect of distinct processes (homogenization, sintering) occurring inside the steel plant on airborne concentrations of PAHs and selected elements was provided. The impact of emissions from the steel plant “core” on the surrounding area was observed at receptor sites downwind to it. Moreover, the extent of the effectiveness of mitigation measures, partially applied at the moment of study’s beginning, was demonstrated by mean and peak pollutant concentrations at all receptor sites up to one order of magnitude lower than those documented prior to 2012.

## 1. Introduction

Numerous epidemiologic studies extensively highlighted the negative impact on human health due to airborne Particulate Matter (PM) exposure. The chemical composition (e.g., organic and inorganic pollutants), along with morphological characteristics of airborne particles are the relevant factors associated with an increased incidence of adverse effects on human health such as pulmonary and cardiovascular diseases [1]. Polycyclic Aromatic Hydrocarbons (PAHs) are ubiquitous organic compounds listed as environmental priority pollutants by the US Environmental Protection Agency (EPA) due to their proven or potential carcinogenic and/or mutagenic properties [2]. PAHs congeners may be present in both gaseous and particulate form and the partitioning between the two phases depends on their physico-chemical characteristics (i.e., vapor pressure, molecular weight) and the atmospheric conditions as well. PAHs of concern for their intrinsic toxicity (e.g., benzo[a]pyrene) are demonstrated to be predominantly adsorbed onto PM fine fraction (PM_2.5_). They mainly originate from the incomplete combustion of organic matter such as coal, coke, gasoline and biomass (e.g., pyrogenic sources), and from the use of fossil fuels (e.g., petrogenic sources). Once emitted from the sources, PAHs may undergo chemical transformation due to both gas-phase and heterogeneous reactions with oxidant species such as ozone, nitrogen oxides, hydroxyl and nitrate radicals. As a result, nitrated and oxygenated derivatives are formed, namely nitro-PAHs (NPAHs) and oxy-PAHs (OPAHs) [3]. In addition to parent-PAHs chemical transformation, NPAHs and OPAHs may be directly emitted by anthropogenic activities involving combustion processes. In recent years, the growing interest among scientists on NPAHs and OPAHs, along with their parent PAHs, based on the knowledge progressively acquired on toxicological implications, resulted in intensive investigations in urban and industrial atmospheres addressed to elucidate on their occurrence and fate [4,5,6,7]. Intensive investigations on PM chemical composition in industrialized and high-density urban areas were also addressed, over the years, for the detection and quantification of trace elements [8]. Special attention has been paid to specific elements such as Manganese (Mn), Lead (Pb) and Cadmium (Cd), which were demonstrated to impair negative effects on human health due to chronic exposure e.g., nervous system damage, cardiovascular and lung diseases [9]. Similarly to PAHs, elements of concern may be emitted by industrial sources as a result of combustion processes or by vehicular traffic. Additionally, they originate from activities involving movement of raw mineral materials or due to dust resuspension. It appears clear, therefore, that there is a need for PM chemical characterization in terms of the aforementioned micro-pollutants and for the identification of emission sources and their contributions in order to both assess the potential impacts on the exposed population and to develop effective air pollution control and remediation strategies [10]. It is an issue of concern and a priority for general public health especially in highly industrialized cities where vast and complex industrial areas are located near urban settlements [11,12].

### 1.1. State-of-the-Art of Scientific Studies in Taranto

Taranto city (40°28′ N 17°14′ E) is ranked as third in the list of high density population cities in Southern Italy. It has been recognized as an Italian site of high environmental risk and included in the list of high priority polluted sites of national interest due to the presence of an extended and complex industrial area nearby the urban settlement. The industrial area is characterized by the coexistence of several anthropogenic activities that, from greater to lower extent, significantly affect the environmental compartment ”air”: the biggest European steel plant, a refinery, the most important harbor in Southern Italy, a cement plant and a naval shipbuilding industry. The evaluation of air quality in Taranto and surrounding areas has been in the last two decades a priority for Italian competent authorities and subject of in-depth investigation for air scientists and epidemiologists with the purpose to identify critical issues and estimate the potential impact on the exposed population. Previous investigations have indeed highlighted that emissions coming from the multiple sources present in the vast industrial area of Taranto synergistically and negatively affected air quality in terms of airborne pollutants such as PAHs and heavy metals [13,14,15,16,17,18,19,20]. The first investigation in the urban area of Taranto was carried out in 2004 by Chiari et al. and was aimed at the identification of the main emission sources affecting the airborne concentrations of elements [13]. High temporal resolution sampling campaigns were performed collecting hourly PM_10_ and PM_2.5_ samples at two receptor sites: one urban site adjacent to the industrial area and one background site (7 km North of Taranto). The highest hourly concentrations of most of the investigated elements were measured at the urban site, prevalently downwind to the industrial area, and for this reason are subjected to a heavy pollution flux. More specifically, peak hourly concentration of Fe and Mn up to 15 µg/m^3^ were measured at the urban site when downwind to the industrial area (wind direction North-West). Source apportionment by Absolute Principal Component Analysis (PCA) applied to both coarse and fine PM fractions datasets highlighted that the main contributions to Fe, Mn, Cu, Zn and Pb airborne concentrations measured at the urban receptor site were related to industrial activities and vehicular traffic. A tailored study to assess the impact of the steel plant activity on air quality of the nearby urban settlement of Taranto in terms of airborne concentrations of PAHs ad elements was performed by Amodio et al. between 2005 and 2006 [14]. PM_10_ and PM_2.5_ sampling campaigns were simultaneously carried out at two receptor sites, one industrial and one urban, considered of interest due to their relative position with respect to the steel plant. The highest PM_10_ and PM_2.5_ concentrations, measured at both receptor sites, occurred under anemometric conditions promoting the transport of pollutants from the steel plant. Similar considerations were made for PAHs and Fe, strengthening the hypothesis of a common emission source placed inside the industrial area. The diagnostic ratio Indeno[1,2,3-cd]pyrene/Benzo[g,h,i]perylene (IP/BgP) provided further evidence of the main contribution of the industrial activities on PAHs concentrations at both the investigated sites (Ip/BgP equal to 0.97 in agreement with reference values in literature). Between the same years (2005 and 2006), a study aiming to investigate the correlation between meteorological parameters and emission sources and their effect on PAHs concentrations in two cities of Apulia Region e.g., Bari and Taranto was carried out by Amodio et al. [15]. The study showed that in Taranto, unlike in Bari, specific anemometric conditions, e.g., wind blowing from the North, exerted a significant effect on PAHs concentrations measured at the urban receptor sites. This evidence took into account that the main source of PAHs was localized in the industrial area (the integrated steel plant). Another comparative study was published in 2007 by Bruno et al. [16] with an aim to identify the main contributions to airborne PAHs concentrations. The PM sampling campaigns were carried out in 2006 at a typical urban site in Bari (on a heavy traffic road) and at an ”industrial” site in Taranto (placed in the closest urban settlement to the steel plant). The PM chemical characterization revealed that in Taranto the mean benzo[a]pyrene concentration derived on monthly scale was 2.05 ± 2.90 ng/m^3^, one order of magnitude higher than that determined in Bari. This study, moreover, highlighted that, in addition to ducted emissions, a non-negligible contribution to airborne pollutant concentrations has to be referred to fugitive emissions from the industrial plants due to the leaks occurring during combustion processes and/or as a result of specific mechanical activities e.g., raw materials handling. In this regard, in order to assess the impact of both ducted and fugitive emissions from the steel plant on the surrounding areas depending on anemometric conditions (wind speed and direction), PM_10_ and PM_2.5_ sampling campaigns were performed by Amodio et al. in 2010 [17]. Three sampling sites around the steel plant were carefully chosen, obtaining a triangulation of the vast source. The integration of PAHs and element concentrations with meteorological parameters showed that highest concentrations of PAHs, Fe, Mn and Zn at each receptor site were measured when downwind from the steel plant. PCA allowed for discrimination of four different emission sources in the area under investigation and estimate their contributions: coke ovens and mineral park were revealed to be the sources that mostly contributed to the measured concentrations of PAHs and elements such as Fe, Mn and Zn, respectively. Further investigations were carried out by Di Gilio et al. in 2011 with the aim to deepen the findings previously documented [18]. For this purpose, an intensive monitoring campaign was performed over six months by collection of PM_10_ samples at seven receptor sites placed in residential settlements at close proximity to the industrial area and in the city center. PAHs concentrations were determined on the collected 1139 PM_10_ samples and integrated with meteorological parameters. Source apportionment analysis by data processing with PCA and Positive Matrix Factorization (PMF) methods identified two sources that mostly affected PAHs concentrations, e.g., industrial source and vehicular traffic, highlighting at the same time that the estimated contribution of the industrial source on PAHs concentrations was the highest at all the receptor sites, even those further away from the steel plant. Finally, the processing of PMF outputs by bivariate polar plots showed that the highest B[a]P concentrations occurred when each receptor site was downwind to the steel plant confirming its remarkable impact on the surrounding area. The unique study aimed at the identification and quantification of Nitro-PAHs in both urban and industrial atmospheres of Taranto was carried out in 2011 by Tutino et al. [19]. Nitro-PAHs concentrations were higher at sites at close proximity to the steel plant and decreased with the increasing of the distance from it. The steel plant were revealed to be the common source of PAHs and nitro-derivatives determining high levels of 2-nitrofluorene (up to 2.64 ng/m^3^) at the receptor sites when downwind to it. Therefore, the inclusion of Taranto in the list of sites at ”high risk of environmental crisis” due to the documented (and here summarized) impacts of the industrial emissions along with the epidemiologic studies correlating the exposure of the population with the increased malignant cancer mortality forced the Environment Ministry of Italy to promptly take measures for air pollution mitigation and control [21]. Cohort studies on populations living in proximity of the industrial area over the decade 1998–2008, indeed, reported mortality excesses in both genders for malignant neoplasms such as lung cancer and pancreatic cancer and non-Hodgkin’s lymphoma, along with several cardiovascular, respiratory and digestive diseases, compared with other territories in the Apulia region [22,23]. Through the issue of the Integrated Environmental Authorization in 2012, the steel plant was obliged to reduce ducted emissions and to implement specific abatement strategies to reduce dust emissions e.g., the installation of fog cannons and the covering over the mineral park. It also prescribed the implementation of a high spatial resolution monitoring network consisting of monitoring stations placed inside the steel plant and along its external perimeter. At the moment of the present study (2014) the high spatial resolution monitoring network was implemented. Moreover, the requirement of emissions reduction was respected through the stopping of the activities of one blast furnace.

### 1.2. Objective of the Present Study

The main objective of the present study was to determine airborne concentrations of PAHs, Nitro- and Oxy-PAHs and elements in both industrial and urban areas of Taranto after the issue of the Integrated Environmental Authorization in 2012. For this purpose, a PM_2.5_ sampling campaign was carried out in 2014 at eight receptor sites, two placed in the urban settlement and six included in the newly implemented fence monitoring network of the steel plant. Source apportionment analysis of the collected data by PMF and bivariate polar plots processing was aimed at the identification, estimation and localization of source contributions. To our knowledge, no study has been published so far with the aim to depict the situation in the investigated area after the application of the Italian government measures for air pollution mitigation and control. The novel aspect of the present study is also related to the high spatial resolution of the sampling campaign performed within and along the perimeter of the steel plant in the industrial area of Taranto allowing an accurate discrimination among source contributions. 

## 2. Materials and Methods

### 2.1. Sampling Sites Description

**Figure 1:**
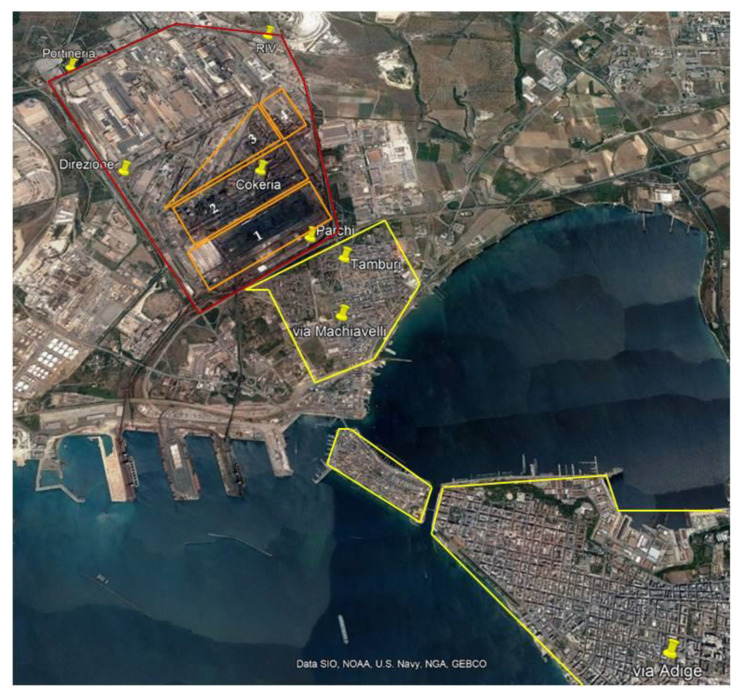
Position of the steel plant (delimited by the red line) with respect to the urban settlements (delimited by the yellow line). Indication of the monitoring stations inside and surrounding the steel plant and the main sectors inside the steel plant (each sector defined by the orange line). Inside the steel plant perimeter (red line): 1: mineral park; 2: coke ovens; 3: blast furnaces; 4: sintering plant.

In order to identify and localize the emission sources within the steel plant area and to evaluate their impacts on the populated surrounding areas, high spatial resolution PM_2.5_ sampling campaign was performed over the period of December 9 to 28, 2014, at eight receptor sites placed inside the industrial and urban areas of Taranto. Six on eight of the receptor sites were environmental monitoring stations included in the fence monitoring network of the steel plant, placed inside the plant and along its perimeter and named Riv, Cokeria, Direzione, Parchi, Portineria and Tamburi (Figure 1). The latter site Tamburi is positioned immediately outside the steel plant perimeter, in the closest urban settlement to the industrial area. 

The other two receptor sites were the industrial site named Machiavelli, placed in a suburban area bordering the southern side of the steel plant at South-South-East, and the urban site Adige placed in the downtown at South-South-West of the steel plant, respectively. As regards the representativeness of the sampling period, based on a preliminary evaluation on PM_10_ and B(a)P annual mean concentrations over the years 2013–2014, after the issue of the Integrated Environmental Authorization, it’s reasonable to assume that the monthly campaign is representative of longer periods and eligible to depict the situation in Taranto after the prescribed reduction of the steel production standards [21].

### 2.2. PM_2.5_ Sampling Methodology 

Daily PM_2.5_ samples collection was performed using a dichotomous low volume sampler, SWAM Dual Sampler (FAI Instruments s.r.l., Roma, Italy). During the monitoring campaign (9–28 December 2014), a total of 160 PM_2.5_ samples were collected onto 47 mm quartz filters (Whatman QM-A, GE Health care) by sampling heads FAI EN 1234.1 operating at a flow rate of 2.3 m^3^ h^−1^. In order to determine PM_2.5_ mass, before and after sampling, quartz filters were properly conditioned for 48 h under controlled environmental conditions (e.g., temperature 22 ± 3 °C and relative humidity 44 ± 7%) and weighed using an analytical microbalance with a sensitivity of 0.0001 mg (Genius Sartorius SE2-F, Sartorius AG, Gottingen, Germany). The entire filters were then cut by means of a stainless steel socket punch resulting in four identical circular sectors: 2/4 sectors were analyzed for PAHs and nitro-, oxy-derivatives determination while one additional sector was used for the characterization of elemental composition. 

### 2.3. PAHs and Nitro-, Oxy-Derivatives Extraction and Analysis by GC-MS/MS

#### 2.3.1. Chemicals

A PAH certified standard mixture (EPA 525 PAH mix A, Supelco: 1 mL vial at 500 µg/mL for each compound in methylene chloride) containing acenaphthylene, fluorene, phenanthrene, anthracene, pyrene, benzo(a)anthracene, chrysene, benzo(b)fluoranthene, benzo(k)fluoranthene, benzo(a)pyrene, dibenzo(a,h)anthracene, benzo(g,h,i)perylene, indeno(1,2,3-cd)pyrene was purchased from Sigma-Aldrich (St. Louis, MO, USA). Authentic standards for nitro- and oxy-PAHs were included in a customized mixture (ML 696: 10 mL vial at 10 µg/mL for each compound in hexane), purchased from Analytical Standard Solutions (A2S, Saint Jean d’Illac, France). The certified standard mixture contained the following individual Nitro- and oxy-PAHs: 5-nitroacenaphthene, 2-nitrofluorene, 9-nitroanthracene, 1-nitropyrene, 3-nitrofluoranthene, 7-nitrobenz(a)anthracene, benzo(a)fluorenone, and 1,2-benzanthraquinone. Moreover, a deuterated PAHs standard mix (EPA 8270 Semivolatile Internal Standard Mix, Supelco: 1 mL vial at 2000 µg/mL for each compound in methylene chloride) containing acenaphthene-d_10_, chrysene-d_12_, 1,4-dichlorobenzene-d_4_, naphthalene-d_8_, perylene-d_12_ and phenanthrene-d_10_ was obtained from Sigma-Aldrich. Hexane and acetone of analytical grade were purchased by Sigma Aldrich. Hexane was used as solvent for the preparation of PAHs and nitro-, oxy-derivatives calibration standards while hexane-acetone mixture for PAHs and derivatives extraction from PM_2.5_ samples.

A PAH certified standard mixture (EPA 525 PAH mix A, Supelco: 1 mL vial at 500 µg/mL for each compound in methylene chloride) containing acenaphthylene, fluorene, phenanthrene, anthracene, pyrene, benzo(a)anthracene, chrysene, benzo(b)fluoranthene, benzo(k)fluoranthene, benzo(a)pyrene, dibenzo(a,h)anthracene, benzo(g,h,i)perylene, indeno(1,2,3-cd)pyrene was purchased from Sigma-Aldrich (St. Louis, MO, USA). Authentic standards for nitro- and oxy-PAHs were included in a customized mixture (ML 696: 10 mL vial at 10 µg/mL for each compound in hexane), purchased from Analytical Standard Solutions (A2S, Saint Jean d’Illac, France). The certified standard mixture contained the following individual Nitro- and oxy-PAHs: 5-nitroacenaphthene, 2-nitrofluorene, 9-nitroanthracene, 1-nitropyrene, 3-nitrofluoranthene, 7-nitrobenz(a)anthracene, benzo(a)fluorenone, and 1,2-benzanthraquinone. Moreover, a deuterated PAHs standard mix (EPA 8270 Semivolatile Internal Standard Mix, Supelco: 1 mL vial at 2000 µg/mL for each compound in methylene chloride) containing acenaphthene-d_10_, chrysene-d_12_, 1,4-dichlorobenzene-d_4_, naphthalene-d_8_, perylene-d_12_ and phenanthrene-d_10_ was obtained from Sigma-Aldrich. Hexane and acetone of analytical grade were purchased by Sigma Aldrich. Hexane was used as solvent for the preparation of PAHs and nitro-, oxy-derivatives calibration standards while hexane-acetone mixture for PAHs and derivatives extraction from PM_2.5_ samples.

#### 2.3.2. Standard Solutions

Six PAHs standard solutions in the concentration range 0.5-12.5 ng/mL (0.5, 1.0, 3.0, 5.0, 8.5 and 12.5 ng/mL) were prepared for calibration. Standard solutions were prepared by serial dilution with hexane of a primary standard at concentration of 600 ng/mL. Similarly, nitro-PAHs and Oxy-PAHs solutions in the concentration range 0.5–10 ng/mL (e.g., 0.5, 1.0, 2.5, 5.0 and 10 ng/mL) were obtained by serial dilution with hexane of a primary standard at concentration of 500 ng/mL. Finally, a standard solution at concentration of 200 ng/mL, containing the deuterated PAHs used as internal standards (ISTD), was prepared in hexane starting from the stock solution.

#### 2.3.3. PAHs and Nitro-, Oxy-Derivatives Extraction Procedure

Microwave-assisted solvent extraction (MAE) procedure was performed to extract PAHs and nitro-, oxy-derivatives from particulate matter collected onto the quartz filters. For this purpose an advanced microwave-based system (MicroSYNTH, Milestone S.r.l., Sorisole, Italy), allowing the simultaneous extraction of 10 samples inside airtight PTFE vessels and under the same experimental conditions, was used. The applied procedure was optimized in a previous published paper and best operating parameters in terms of temperature, extraction volume, time and microwave source power were set for an effective PAHs and derivatives extraction [19]. More specifically, half of each PM_2.5_ filter (2/4 sectors) was immersed into 10 mL of acetone/hexane mixture (1:1) and the extraction was performed for 25 min at 110 °C with microwave source power of 200 W (final vent time of 5 min). The same procedure was applied to all PAHs and nitro-, oxy-derivatives standard solutions by spiking 50 µL onto a half of a quartz blank filter. An aliquot (10 µL) of the deuterated PAHs mix solution was finally added to 1 mL of both standard and real samples extracts. For quality assurance, in each extraction sequence, one PTFE vessel containing the solvent mixture and half blank quartz filter was introduced.

#### 2.3.4. PAHs and Nitro-, Oxy-Derivatives Analysis by GC-MS/MS

PAHs and nitro-, oxy-derivatives characterization was performed analyzing the extracts with an Agilent 7000A GC/MS Triple Quadrupole System (Agilent Technologies, Santa Clara, CA, USA) consisting of 7890A gas chromatograph equipped with a Programmable Temperature Vaporization system (PTV) and a 7000 triple quadrupole mass spectrometer (QQQ) equipped with an inert ion source. Liquid injection was performed by means of MPS2 L automatic liquid sampler (Gerstel GmbH & Co, Mülheim an der Ruhr, Germany), coupled to the GC/MS system, and equipped with a 10 µL syringe. PTV injection method is preferred when high sensitivity is required for the detection of trace pollutants such as PAHs and nitro-, oxy-PAHs in complex environmental matrices where co-elution of matrix interferences is likely to occur [24]. A single tapered deactivated inlet liner (Agilent Technologies Inc, Palo Alto, CA, USA) was installed into the GC injector. Ten micro liters of each final extract was injected into the GC liner in solvent vent mode with a flow vent of 50 mL/min, kept constant for 1 min in order to ensure a quantitative removal of the used solvents (acetone and hexane). The liner was hold at 60 °C for 1.2 min; afterwards the temperature was rapidly increased with a rate of 720 °C/min until the achievement of the final temperature equal to 300 °C. The purge valve was opened and a purge flow of 65 mL/min for 2 min was applied. The separation was performed onto TraceGold TG-17MS GC column (Thermo Fischer Scientific, Waltham, MA, USA), 30 m × 0.25 mm id with 0.25 μm film thickness. The oven program used for the optimal separation was as follows: initial temperature at 30 °C (hold time 2.5 min), then ramp 1: 30 °C/min up to 210 °C, then ramp 2: 5 °C/min up to 280 °C (final hold time 25 min). The total run time was 45 min. High purity helium gas (>99.999%) was used as carrier in constant flow mode at 1.3 mL/min. The mass spectrometer was operated in electron impact ionization (EI) mode (70 eV) in the mass range 50–300 *m*/*z*. GC/MS transfer line and EI ion source temperatures were kept at 300 °C and 290 °C, respectively. The QQQ collision cell He quench gas and N_2_ collision gas were both set at 1 mL/min. GC-MS/MS data were analyzed using Mass Hunter Workstation software Quantitative Analysis v. B.04.00 (Agilent Technologies, Santa Clara, CA, USA). After a preliminary acquisition in SCAN mode for the determination of the retention times, analyte ions were monitored in Multiple Reaction Monitoring (MRM) mode. PAHs and nitro-, oxy-PAHs were acquired separately using two different MRM acquisition sequences, subdivided each in several time segments. Table 1 and Table 2 lists PAHs and nitro-, oxy-PAHs investigated in this study, along with deuterated compounds used as ISTD for quantification. MRM acquisition parameters are therein reported: time segments, retention times (T_R_, min), precursor ions, quantifier ions, qualifier ions and collision energy (CE, eV). PAHs and nitro-, oxy-PAHs were identified by their retention times and quantifier and qualifier ions. Criteria for compounds identification involved matching of the retentions times with those of authentic standards within ±1 min of the expected values. Quantification was performed based on integrated peak area ratio of the quantifier ion and the reference internal standard, acquired in the same time segment. Six levels-based calibration curves were constructed for PAHs within the dynamic concentration range 0.5–12.5 ng/mL. Five levels-based calibration curves were constructed for nitro- and oxy-PAHs within the dynamic concentration range 0.5–10 ng/mL. In both cases calibration curves were obtained by liner regression of the calibration standards responses using three points for each concentration levels (correlation coefficients R^2^ > 0.98 for all the investigated compounds). Limit of detection (LOD) and Limit of Quantification (LOQ) of PAHs along with their nitro-, oxy-derivatives ranged from 0.012 to 0.068 ng/mL (0.004–0.025 ng/m^3^) and from 0.041 to 0.23 ng/mL (0.015–0.083 ng/m^3^), respectively. Finally, due to coelution, anthracene, phenanthrene, benzo[b]fluoranthene and benzo[k]fluoranthene were not allowed to be separately quantified. Therefore anthracene+phenathrene and benzo[b]fluoranthene+benzo[k]fluoranthene sum concentrations were determined.

### 2.4. Elements Extraction and Analysis by ICP-MS

#### 2.4.1. Chemicals and Standard Solutions

A multi-element certified standard mix in 2% nitric acid (concentration 20 mg/L) was purchased by Chemical Research 2000. Six multi-element standard solutions were prepared by dilution of the primary standard with a 0.6% nitric acid solution obtaining the following concentration levels: 0.02, 0.2, 2, 20, 100 and 300 ppbv. The internal standard mix was purchased by CPA Chem and contained eight elements: Scandium (Sc), Germanium (Ge), Rhodium (Rh), Indium (In), Terbium (Tb), Holmium (Ho), Lithium (Li), Terbium (Tb) and Bismuth (Bi) at 10 mg/L. The internal standard solution used for quantification was prepared by the primary standard by dilution with nitric acid at 0.5% resulting in 20 ppbv concentration. Ultrapure grade nitric acid 60% and hydrogen peroxide 30% were obtained from Sigma Aldrich and Carlo Erba, respectively.

#### 2.4.2. Elements Extraction Procedure

One-fourth of each PM_2.5_ quartz filter was digested in 8 mL of nitric acid and 2 mL of hydrogen peroxide solutions in compliance with the requirements of European standard method EN14905:2005 [25]. Elements extraction was performed inside airtight PTFE digestion vessels using an advanced microwave-based system (MicroSYNTH, Milestone S.r.l., Sorisole, Italy) through three steps of a temperature-programmed procedure, summarized as follows: step 1 (heating): from 35 °C up to 180 °C for 20 min at 1200 W; step 2 (heating): from 180 °C up to 220 °C for 20 min at 1220 W; step 3 (cooling): from 220 °C to 35 °C for 20 min. Once the extraction process was completed, the vessels were allowed to cool for several hours and each 10 mL solution was diluted with ultrapure water 18 MΩ/cm (1:1) and safely stored before analysis. For quality assurance, in each extraction sequence, one PTFE vessel containing the mixture of reagents and one-fourth of blank quartz filter was introduced. 

#### 2.4.3. Elements Analysis by ICP-MS

The obtained extracts were analyzed by ICP-MS iCAP Q system (ThermoFisher Scientific, Waltham, MA, USA). Each solution was further diluted 1:30 before injection. The analysis sequences were performed with argon nebulizer flow and plasma gas flow of 1 L/min and 18 L/min, respectively. Elements Fe, Mn, Pb, Ni, Ti, As and Cd were quantified with six-point calibration curves obtained by liner regression of the multi-element calibration standards responses in the dynamic concentration range 0.02–300 ppb. The calibration levels were: 0.02, 0.2, 2, 20, 100 and 300 ppb. The resulting correlation coefficients R^2^ were typically > 0.999. Limit of Quantification (LOQ) for all the investigated elements were in the range 0.23–7.10 ng/mL. Extraction recoveries were previously determined in the optimization process of the analytical technique and varied in the range from 87% to 100%.

### 2.5. Source Apportionment Analysis

PAHs, elements and PM_2.5_ daily concentrations for all the investigated sites along with the meteorological data of the period were used for source apportionment analysis. The meteorological data such as wind speed, wind direction, atmospheric temperature, pressure and relative humidity were downloaded by the Regional Agency for Environmental Protection (ARPA Puglia) website and processed by R-OpenAir wind rose software. Environmental data sets modeling was performed by Positive Matrix Factorization (PMF). PMF is a multivariate factor analysis technique able to determine, through the non-least square regression, the best fit source fingerprints (e.g., factors) and contributions, starting from input data sets of pollutant concentrations at receptor sites and associated uncertainties [26]. In this study, data sets modeling was performed using EPA PMF v5.0 software. More specifically, the concentration and uncertainty input matrices were constructed from PAHs, elements and PM_2.5_ data and the input variables were classified according to Signal-to-Noise (S/N) criteria [27,28]. Most reasonable PMF solutions were explored based on maximum Individual column Mean (IM), maximum Individual column Standard deviation (IS), goodness of fit parameter (Q-value) and for multiple values of the peak coefficient (Fpeak, between −0.1 and +1.0, with step of 0.2). Finally, PAHs and elements concentrations and the PMF solutions were further processed by bivariate polar plot analysis with Open Air R-package software [29,30]. Polar plots are radial diagrams where the pollutant concentration with respect to the wind direction and speed is visualized, providing emission sources characteristic information in the investigated area. Polar plots were obtained subdividing wind speed and direction data in a sequence of direction-speed sectors, consisting of intervals with 10° width and wind speed between 0 and 30 m/s.

## 3. Results and Discussion

An overall discussion of the results obtained from the chemical characterization of PM_2.5_ samples and on the outputs of source apportionment analysis is herein reported.

### 3.1. PAHs and Elements Results

**Figure 2:**
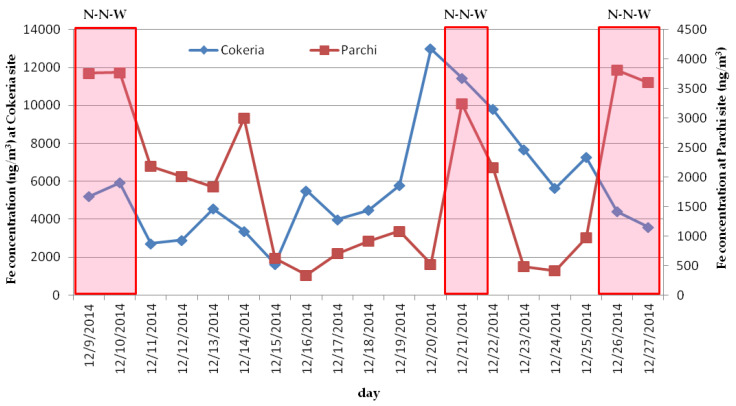
Fe daily concentration trends at Cokeria and Parchi sites from 9 to 27 December 2014 (last day of sampling period missing) and prevailing wind direction in selected days.

PAHs and element concentrations investigated in this study, over the sampling period (9–28 December 2014) and for all the receptor sites, are listed in Table 3 as minimum, maximum and mean values (ng/m^3^). Regarding PAHs, dibenzo[a,h]anthracene concentrations resulted to be lower than the LOQ in the majority of the collected samples (>95%) and, for this reason, are not reported in the dedicated table. Minimum, maximum and mean values of PM_2.5_ concentrations for each site, expressed as µg/m^3^, are also therein reported. Preliminarily to the discussion of the obtained results, it is necessary to highlight that, based on meteorological data processing, the sampling period was overall characterized by wind calm (low wind speed) with the exceptions of days 9, 10, 12 and 21 December characterized by high wind speed and direction North-North-West and the day 16 December by high wind speed and direction South-South-West. During the sampling period, the mean PM_2.5_ concentration at Cokeria site was equal to 47.4 µg/m^3^, higher than those determined at all the other receptor sites falling in the concentration range from 11.2 µg/m^3^ (Portineria) to 21.9 µg/m^3^ (RIV). From the comparison among the receptor sites, it is possible to observe that mean concentrations of the investigated PAHs and elements at Cokeria site (the closest site to the coke ovens of the steel plant) were significantly higher than those measured at all the other sites, up to one order of magnitude (Table 3). In more detail, benzo[a]pyrene (B[a]P) mean concentration measured at Cokeria site over the sampling period was equal to 6.3 ng/m^3^ while, for all the other sites, it ranged from 0.43 ng/m^3^ (Portineria) to 0.74 ng/m^3^ (RIV). Moreover, during the sampling period at Cokeria site, exceedances of B[a]P annual target value equal to 1 ng/m^3^ were observed: in 8 on 20 days of the sampling campaign the B[a]P daily concentration reached values above the target value of 1 ng/m^3^ ranging from 1.3 ng/m^3^ (slightly above) to 32.7 ng/m^3^ (one order of magnitude higher). B[a]P mean concentrations observed at the receptor sites placed in the urban settlement were comparable, with values at Machiavelli and Adige sites equal to 0.44 and 0.50 ng/m^3^ respectively. From the comparison with findings reported in previous studies and obtained from winter sampling campaigns carried out prior the issue of the Integrated Environmental Authorization (2012), it possible to point out that B[a]P concentrations in Taranto urban area obtained in the present study are up to one order of magnitude lower than those previously documented [14,16]. More specifically, B[a]P concentrations averaged on two different sampling periods within autumn-winter seasons in 2005–2006 at one urban site in Tamburi settlement (close to Machiavelli site in this study) were 0.96 and 2.04 ng/m^3^. Moreover, exceedances of B[a]P annual target value equal to 1 ng/m^3^ were frequently observed at the urban site during the whole sampling period (in 12 of 40 days of the sampling campaign) with peak concentrations (1.49–8.7 ng/m^3^) during the days when the site was downwind to the industrial area (therefore to the steel plant) [14]. On the contrary, in the present study, exceedances of B[a]P target value at Machiavelli site were not observed. Therefore, on the basis of the data available in literature, this comparison underlines that, following the air pollution mitigation measures partially applied in 2012, B[a]P mean concentrations as well as the number of exceedances in the urban area of Taranto were significantly reduced due to the reduced production standards of the steel plant. This evidence is also confirmed by the preliminary evaluations made by Trizio et al., 2016 that highlighted the decreasing trend of both PM_10_ and B[a]P annual mean from 2009 to 2014 on the basis of daily data collected from the Regional Air Quality Network in Taranto (B[a]P concentrations were collected by high resolution instrumentation of the network monitoring stations) [21]. In this study, moreover, it was observed the decrease in airborne concentration of all the investigated PAHs with the increasing in the distance from the Cokeria site where the highest concentrations were measured (Table 3). A similar behavior was observed for some elements. In this regard, it is interesting to underline that mean concentrations of elements such as Fe, Mn and Zn at Cokeria site resulted to be higher than those observed at Parchi site (the closest receptor site to the mineral park) suggesting that the core area of the steel plant including the blast furnaces and the coke ovens represents the main source of the aforementioned elements in the area. More specifically, among the investigated elements, Fe was the most abundant with a mean concentration at Cokeria site equal to 5.7 µg/m^3^, approximately three times higher than that measured at Parchi site and one order of magnitude higher than those at all the other receptor sites in the investigated area (Table 3). In the urban settlements, at Machiavelli and Adige sites, mean Fe concentrations were significantly lower than those reported in previous investigations (prior 2012) [14,17]. It appears also evident that peak Fe concentrations, observed when the urban sites were downwind to the industrial area, were in this study one order of magnitude lower than those related to prior-2012 period confirming the effect of mitigation measures in reducing the impact of industrial activities on the urban area. The potential correlation among B[a]P and specific elements Fe, Mn, Zn and Pb at Cokeria site was studied by deriving correlation coefficients from daily concentrations over the sampling period. The obtained correlation coefficients highlighted that high correlation was: between Fe and Mn (correlation coefficient: 0.9), between Pb and Zn (correlation coefficient 1.0), between Pb and B[a]P (correlation coefficient 0.9) and between Zn and B[a]P (correlation coefficient 0.9). On the contrary, no correlation was found between B[a]P and Fe and B[a]P and Mn (correlation coefficients equal to 0.1). This allowed to speculate on the coexistence and proximity inside the industrial area of two different emission sources. In this regard, an in-depth analysis of industrial processes occurring in different specialized areas inside the steel plant was needed in order to strengthen this hypothesis. Inside an integrated steel plant, the production cycle is based on the agglomerate formation consisting of several processes: (1) homogenization and blending of primary raw materials; (2) sintering; (3) cooling and final treatment of the agglomerate [31]. The first process, e.g., homogenization and blending process of primary raw materials, is performed with the specific purpose to improve the mechanical properties of the steel (e.g., strength, hardness and ductility). It necessarily involves the transport of primary raw materials on conveyor belts starting from the mineral park, the homogenization and blending of iron ore and specific additives such as limestone and Mn and finally the loading of the obtained material onto the agglomeration machine for the successive sintering process. The sintering process, instead, represents the heat treatment of the agglomerate allowing to obtain a material characterized by the proper granulometry for the transfer in the blast furnaces. During the sintering process emission of elements such as Pb and Zn may occur. Therefore, taking into account that at Cokeria site high correlation was observed: (a) between selected elements e.g., Fe correlated with Mn, Pb correlated with Zn; (b) between PAHs and selected elements e.g., B[a]P correlated with Pb and Zn; and starting from the observation that mean concentrations of PAHs and elements at Cokeria site were significantly higher than those determined in other receptor sites and in particular at Parchi site (located at close proximity to the mineral park), it is possible to preliminarily speculate on the typology of emission sources. Fe with Mn, Zn with Pb and PAHs are emitted by two distinct sources, both located in a delimited area inside the steel plant and close to Cokeria site. The two sources are therefore supposed to be related to the homogenization and blending processes (source 1: Fe and Mn) and to the sintering process (source 2: Zn, Pb and PAHs). In order to confirm this hypothesis, the comparison of Fe daily concentration trends over the investigated period at both Cokeria and Parchi sites is reported in Figure 2. 

It is possible to point out that the highest daily Fe concentrations at Parchi site were observed when downwind with respect to Cokeria site e.g., during the days characterized by wind direction North-North-West (N-N-W) able to promote the transport of pollutants from the area at close proximity to Cokeria site to the receptor site Parchi (9, 10, 21, 26 and 27 December).

Since emission sources show specific PAHs concentration profile, attention has been also paid on PAHs diagnostic ratios, more specifically B[a]P/B[g,h,i]P, IP/(IP+B[g,h,i])P and IP/B[g,h,i]P [15,18,32,33,34,35,36]. Mean values of B[a]P/B[g,h,i]P, IP/(IP+B[g,h,i])P and IP/B[g,h,i]P diagnostic ratios calculated for all the receptor sites over the sampling period and relative ranges reported in literature with respect to the typology of emission source are reported in Table 4. The mean values for each selected diagnostic ratio were not affected by a significant variability from one to another receptor site within the area under investigation. In more detail, B[a]P/B[g,h,i]P ranged from 0.68 (Machiavelli) to 0.97 (Cokeria), IP/(IP+B[g,h,i])P ranged from 0.47 (Tamburi) to 0.52 (RIV) and finally IP/B[g,h,i]P ranged from 0.90 (Tamburi) to 1.06 (RIV). The obtained mean values for all the diagnostic ratios, moreover, were within the range or close to single values reported in literature and typical of ”coke combustion” and ”coal burning” emission sources (Table 4). This evidence suggests that, during the sampling campaign, the predominant source of PAHs affecting their concentrations at both industrial and urban sites in Taranto was the coke combustion. 

It is also relevant to underline that IP/B[g,h,i]P and IP/(IP+B[g,h,i])P ratios, affected by the lowest variability over the sampling period at Cokeria site (from 0.94 to 1.07 and from 0.48 to 0.52 respectively), reached the daily maximum values at all the other investigated receptor sites when downwind to the industrial area confirming the relevance of the contribution of the integrated steel plant. Finally, Table 5 summarizes the latest published studies focused on the evaluation of the impact of steelworks-related industrial emissions on the airborne PAHs and elements concentrations in world’s leading countries in the steel production. Criteria for the selection of the studies were the typology of the industrial site under investigation (characterized by the presence of a vast steelworks facility representing the main industrial source in the area) and the applied methodological approach. For comparison, the results from the present study and the limit values from International Guidelines and both EU and National Air Quality Standards are reported as well (grey section of the table). From the comparison with the relevant literature, it is possible to observe that PM_2.5_ concentrations measured nearby the investigated (and here reported) steelworks facilities worldwide are of the same order of magnitude ranging from the lowest value of 11.2 µg/m^3^ (Taranto city, Italy) to the highest value of 81.22 µg/m^3^ (Anshan City, China), with the only exception of Port Talbot (United Kingdom, UK) where the lowest concentrations are documented (range 6.5–9.2 µg/m^3^). For the considered EU countries (with the only exception of UK) exceedances of PM_2.5_ limit value on yearly basis (25 µg/m^3^) established by the EU Legislation are observed. The most remarkable result, however, is related to the industrial site in Anshan City where the measured PM_2.5_ concentration equal to 81.22 µg/m^3^ is significantly higher than the National 1-year limit value (35 µg/m^3^, Air Quality Standard of China). As regards PAHs, the literature summary reveals that benzo(a)pyrene airborne concentration (maximum value equal to 82.1 ± 129 ng/m^3^) measured in the vicinity of the integrated iron and steel plant in India (location not specified) was significantly higher than those reported in other studies carried out both in EU and extra-EU countries (China) where the annual limit value of 1 ng/m^3^ is in force. Finally, with specific regard to the elements considered as markers of the complex steel production process e.g., Mn, Fe, Zn and Pb, the comparison allows to point out that the industrial site in Hangzhou City (China) was generally characterized by high concentration levels: the highest for Pb (161.6 ± 33.0 ng/m^3^) and Zn (729.2 ± 261.2 ng/m^3^) if compared with the other studies. Significant values for Fe concentrations were also observed in Germany (17,310 ng/m^3^) and in Italy (present study, 5727 ng/m^3^). No exceedances for regulated elements (Pb, As, Cd and Ni) were observed in all EU countries. On the contrary, the As concentration reported for the industrial site in Hangzhou City (16.8 ± 3.4 ng/m^3^) was one order of magnitude higher than the limit value of 6 ng/m^3^ established by the Air Quality Standards, currently in force in China, while remarkable exceedances of Cd and Ni concentrations may be underlined for the industrial site in Mangalore (India) where the measured levels (Cd: 90 ± 40 ng/m^3^; Ni: 1820 ± 110 ng/m^3^) were significantly higher, one-two order of magnitude, than the WHO limit values of 5 ng /m^3^ and 25 ng/m^3^ respectively. This overview therefore highlights that, although the attention on the issue is very high both at International and National level worldwide, tailored investigations nearby the steelworks-related industrial sites in order to evaluate the potential impact of the emissions on the surrounding areas and to verify the compliance with the standards in force along with the development and implementation of new technologies and air pollution abatement strategies are always needed in view of both human health and environment protection.

### 3.2. Considerations on Nitro- and Oxy-PAHs 

Although the presence of nitro- and oxy-PAHs was detected in most of the collected samples, their concentrations at the investigated sites resulted to be generally lower than or close to the LOQ of the applied analytical methodology. Therefore, the investigation of the samples in terms of nitrated and oxygenated PAHs did not provide any useful information neither for the comprehension of atmospheric processes (e.g., dispersion, transport) affecting the spatial distribution of pollutants from the sources to the receptor sites nor for the source apportionment. An attempt to explain this finding can be made taking into account two different aspects. In several studies focused on the determination of airborne concentrations of nitrated and oxygenated PAHs in both urban and industrial sites, it has been extensively highlighted that nitro-and oxy-PAHs concentrations are 2 up to 3 orders of magnitude lower than their parent-PAHs concentrations [50,51]. Therefore, it is likely to occur that the determination of nitro-and oxy-PAHs concentrations is limited by the sensitivity of the applied analytical methodology [52]. Moreover, it is reasonable to assume that the reduction of the productive standards the steel plant was forced to after the issue of the Integrated Environmental Authorization on 2012 resulted in lower PAHs and nitro- and oxy-derivatives concentrations in the investigated area compared with the prior-2012 period [14,16]. 

### 3.3. Statistical Analysis Results 

**Figure 3:**
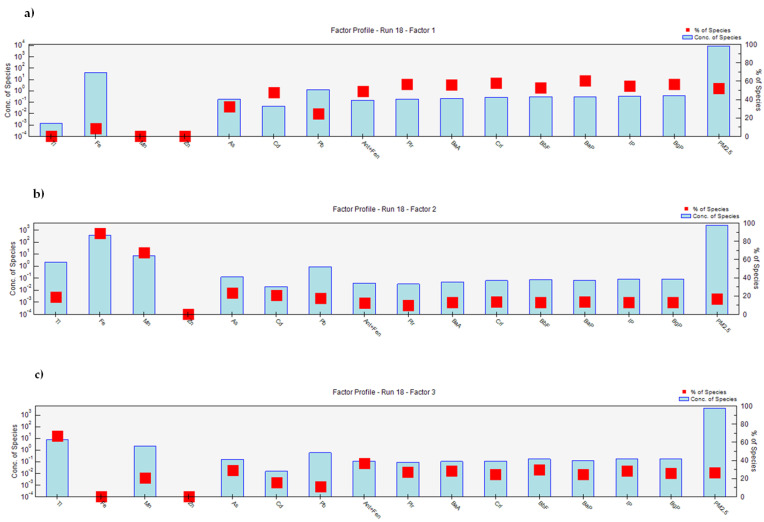

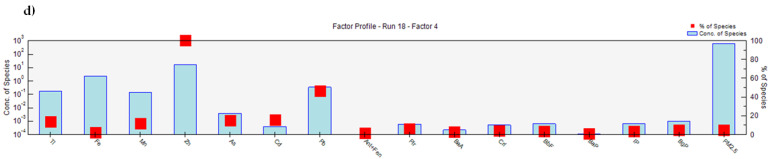
Pollutants concentration and percentage for each identified factor: (**a**) “coke ovens”; (**b**) “homogenization process/mineral park”; (**c**) “traffic”; (**d**) “sintering”.

In order to quantify source contributions and to localize them in the investigated area, the collected data (e.g., PM_2.5_, PAHs and elements daily concentrations) were processed by PMF model and the resulting outputs further treated by polar plots. Four distinct factors were identified, as it is possible to observe from the factor profiles reported in Figure 3. They may be described as follows: one factor characterized by high percentage of all the investigated PAHs and likely to be related to coke ovens (factor 1: “coke ovens”); one factor characterized by high percentages of specific elements such as Fe and Mn and mainly related to the homogenization process of raw materials and loading of the agglomerate in the blast furnaces. The contribution from the mineral park due to particle resuspension is also taken into account (factor 2: “homogenization/mineral park”); one factor characterized by the elements Ti, Cd and As and by PAHs and attributable to the traffic source (factor 3: “traffic”); and finally, one factor characterized by high percentages of Zn and Pb and related to the sintering process in addition to the combustion in the blast furnaces (factor 4: “sintering”).

**Figure 4:**
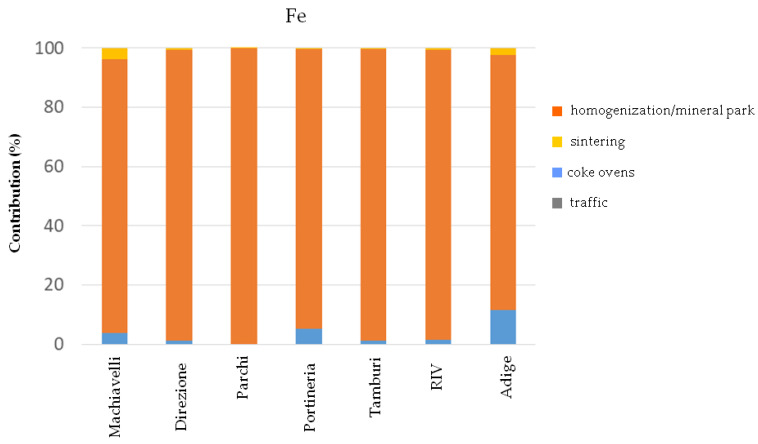
Percentage contribution of factors on Fe concentrations at the receptor sites.

**Figure 5:**
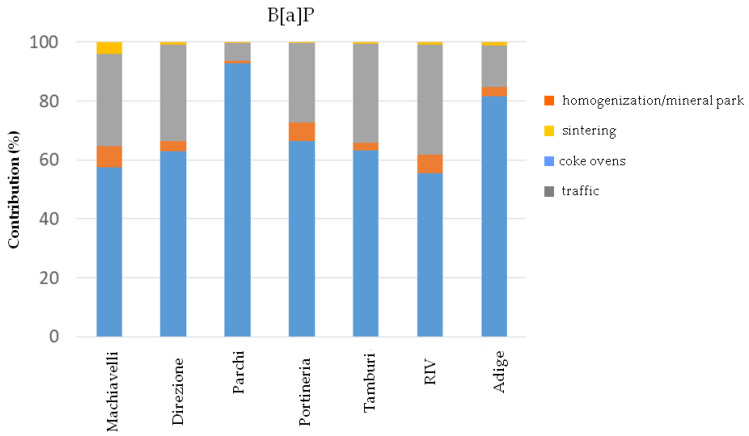
Percentage contribution of factors on B[a]P concentrations at the receptor sites.

**Figure 6:**
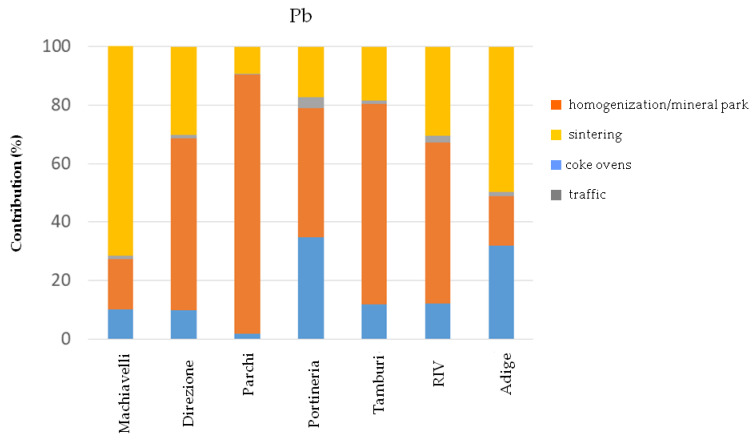
Percentage contribution of factors on Pb concentrations at the receptor sites.

Figure 4, Figure 5 and Figure 6 show the percentage contributions of each identified factor on atmospheric concentrations of Fe, B[a]P and Pb observed at the receptor sites, respectively. The analysis of the percentage distribution of the factors among the sites provided further evidence that the predominant contribution affecting Fe concentrations at all the receptors, including the urban site Adige approximately 10 km far from the steel plant, is the result of the homogenization process of raw materials and loading of the agglomerate in the blast furnaces with the resuspension of particles in the mineral park (Figure 4). Moreover, the higher Fe concentrations daily measured at Cokeria site with respect to Parchi site (the latter closer to the mineral park area) suggest that both homogenization and loading activities contribute to a greater extent on atmospheric Fe concentrations if compared to the resuspension process (Table 4). Regarding, instead, PAHs and more specifically B[a]P the most relevant contribution of their atmospheric concentrations is represented by the combustion process occurring in the coke ovens, regardless of the typology of the receptor site (e.g., industrial or urban). A secondary contribution, but not negligible, is represented by the vehicular traffic (Figure 5).

Finally, the measured atmospheric Pb concentrations were mainly determined by two factors e.g., the sintering process and particle resuspension in the mineral park, as shown in Figure 6. These findings are also confirmed by the bivariate polar plots obtained by PMF outputs processing. 

**Figure 7:**
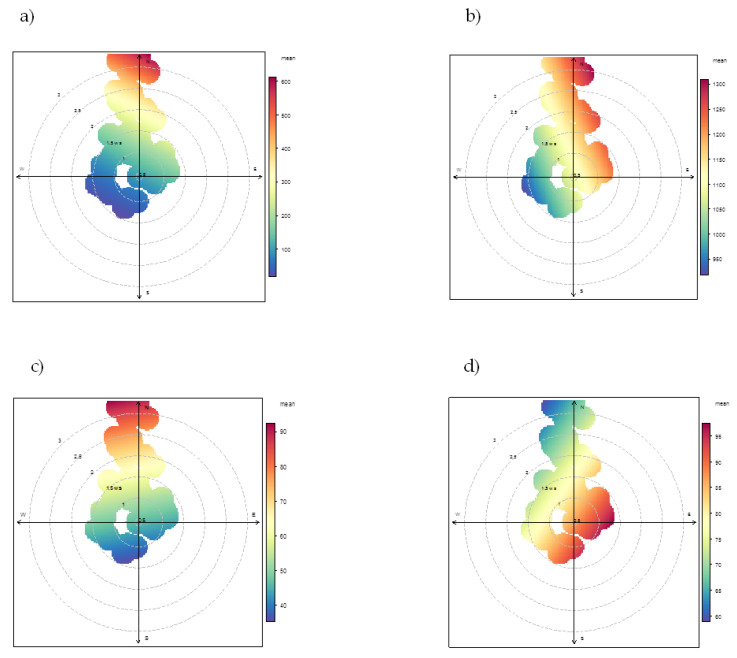
Polar plots of PMF outputs for the receptor sites downwind to Cokeria site: Parchi, Machiavelli, Tamburi and Adige. Factors: (**a**) “coke ovens”; (**b**) “homogenization/mineral park”; (**c**) “sintering”; (**d**) “traffic”.

Figure 7 shows the polar plots obtained by processing of the data collected at sites downwind to the steel plant e.g., Parchi, Tamburi, Machiavelli and Adige. Polar plots of factors 1, 2 and 4, associated to the sources ”coke ovens”, ”homogenization/mineral park” and ”sintering” respectively, show that their highest contributions occurred during days when the prevailing wind direction was North. The wind blowing from the North, indeed, promoted the transport of pollutants (elements and PAHs) from specific sources inside the steel plant to the considered receptor sites. On the contrary, the contribution of the factor 3 associated to the vehicular traffic is also detectable under conditions of wind calm.

## 4. Conclusions

This study was performed in an effort to identify emission sources and estimate their contributions to airborne concentrations of high concern pollutants in Taranto city (a recognized site of high environmental risk in Southern Italy characterized by a vast and complex industrial area nearby the urban settlement) after the implementation of certain strategic measures for air pollution mitigation and control issued by the Italian Environment Ministry in 2012. A tailored investigation was carried out performing a PM_2.5_ sampling campaign from 9 to 28 December 2014 with high spatial resolution within and along the external perimeter of the biggest integrated steel plant in Europe. The chemical characterization of the collected PM_2.5_ samples in terms of selected PAHs, nitro- and oxy-PAHs and elements was performed. Integration of the spatial distribution data of the selected pollutants with meteorological parameters (wind speed and direction), as well as the source apportionment analysis of the collected data by PMF and bivariate polar plots was aimed at the identification, localization and estimation of source contributions. The airborne concentrations of the investigated pollutants at the receptor sites over the sampling period and the meteorological parameters allowed to speculate upon the coexistence and proximity of two different source contributions within the integrated steel plant perimeter affecting Fe, Mn, Zn and PAHs airborne levels. The two contributions were supposed to be related to certain activities; more specifically, the homogenization and blending processes (for Fe and Mn) and the sintering process (for Zn, Pb and PAHs). Source apportionment analysis allowed to identify four main sources in the industrial and urban areas of Taranto: “coke ovens” for all the investigated PAHs; “homogenization/mineral park” for the elements Fe and Mn; “traffic” for the elements Ti, Cd and As and for PAHs; and finally “sintering” for the elements Zn and Pb. The treatment of high spatial resolution data, therefore, provided evidence that selected elements such as Fe and Mn, historically linked to the handling of raw materials in the mineral park of the steel plant and to the particle resuspension, are also emitted into the atmosphere as a result of the homogenization process and loading of the agglomerate in the blast furnaces. Moreover, the comparison with the available literature data on air pollution levels in the investigated area allowed to highlight that, following the air pollution mitigation measures partially applied in 2012, Fe and B[a]P mean concentrations as well as the number of B[a]P exceedances of the annual target value (1 ng/m^3^) in the urban area of Taranto at the end of 2014 were significantly reduced. It is reasonable to assume that this evidence is associated with the efficient strategies aimed to the reduction of the steel plant production standards. Finally, it is important to underline that the high spatial resolution of the sampling campaign performed in this study, benefiting of the fence monitoring network of the steel plant implemented after 2012, allowed us to obtain an accurate discrimination among source contributions. 

## Figures and Tables

**Table 1:** List of PAHs and ISTD. MRM acquisition parameters: time segments, retention times (T_R_, min), precursor ions, quantifier and qualifier ions, collision energy (CE, eV). CAS numbers are also provided.

Compound	CAS #	ISTD	T_R_ (min)	Precursor Ion (*m*/*z*)	Quantifier Product Ion (*m*/*z*)	Qualifier Product Ion (*m*/*z*)	CE (eV)
**Time segment 1: 11.6 min**							
fluorene	86-73-7		9.7	166	165	115	25
phenanthrene	85-01-8		11.5	178	178	152	15
anthracene	120-12-7		11.5	178	178	152	15
phenanthrene-d_10_	1517-22-2	x	11.6	188	188	160	15
**Time segment 2: 21.0 min**							
pyrene	129-00-0		16.0	202	202	176	25
benzo[a]anthracene	56-55-3		20.6	228	228	226	15
chrysene	1719-03-5		20.9	228	228	226	15
chrysene-d_12_	1719-03-5	x	21.0	240	240	238	15
**Time segment 3: 30.0 min**							
benzo[b]fluoranthene	205-99-2		26.0	252	252	250	25
benzo[k]fluoranthene	207-08-9		26.2	252	252	250	25
benzo[a]pyrene	50-32-8		29.1	252	252	250	25
perylene-d_12_	1520-96-3	x	30.0	264	264	260	25
**Time segment 4 45.0 min**							
indeno[1,2,3-cd]pyrene	193-39-5		40.3	276	276	274	25
dibenzo[a,h]anthracene	53-70-3		40.8	278	278	276	15
benzo[g,h,i]perylene	191-24-2		45.0	276	276	274	25

**Table 2:** List of nitro- and oxy-PAHs and ISTD. MRM acquisition parameters: time segments, retention times (TR, min), precursor ions, quantifier and qualifier ions, collision energy (CE, eV). CAS numbers are also provided.

Compound	CAS #	ISTD	T_R_ (min)	Precursor Ion (*m*/*z*)	Quantifier Product Ion (*m*/*z*)	Qualifier Product Ion (*m*/*z*)	CE (eV)
**Time segment 1: 16.4 min**							
phenanthrene-d_10_	1517-22-2	x	11.6	188	188	160	15
5-nitroacenaphtene	602-87-9		14.6	152	151	126	15
2-nitrofluorene	607-57-8		15.7	165	165	115	15
9-nitroanthracene	602-60-8		16.4	223	223	193	5
**Time segment 2: 21.0 min**							
9-nitrophenanthrene	954-46-1		17.3	165	165	115	15
benzo[a]fluorenone	479-79-8		19.3	230	230	202	5
chrysene-d_12_	1719-03-5	x	21.0	240	240	238	15
**Time segment 3: 23.1 min**							
3-nitrofluoranthene	892-21-7		22.4	247	247	217	5
3-nitrofluoranthene-d9	892-21-7	x	22.5	247	247	217	5
1,2-benzanthraquinone	2498-66-0		23.1	258	258	230	5
**Time segment 4: 30.0 min**							
1-nitropyrene	5522-43-0		23.8	201	200	151	25
7-nitrobenz[a]anthracene	20268-51-3		27.2	215	215	189	15
perylene-d_12_	1520-96-3	x	30.0	264	264	260	25

**Table 3:** PAHs, elements and PM_2.5_ concentrations: minimum, maximum and average values for all the investigated sites over the period 9–28 December 2014.

	Site MACHIAVELLI			Site DIREZIONE			Site PARCHI			Site PORTINERIA			Site COKERIA			Site TAMBURI			Site RIV			Site ADIGE		
	Min	Max	Mean	Min	Max	Mean	Min	Max	Mean	Min	Max	Mean	Min	Max	Mean	Min	Max	Mean	Min	Max	Mean	Min	Max	Mean
***PAHs* (ng/m^3^) **																								
Fluo	0.73	4.30	2.36	<LOQ	<LOQ	<LOQ	0.21	0.40	0.30	0.17	0.36	0.25	0.64	8.60	2.48	0.21	0.66	0.42	0.37	0.57	0.45	0.23	0.47	0.32
Anth + Phen	0.22	0.30	0.26	0.24	0.52	0.33	0.23	0.30	0.26	0.22	0.31	0.25	0.81	2.41	1.31	0.24	0.34	0.29	0.50	0.57	0.53	0.23	0.33	0.27
Pyr	0.23	0.43	0.34	0.22	0.45	0.30	0.22	0.44	0.27	0.20	0.32	0.24	0.48	3.21	1.44	0.22	0.54	0.33	0.44	0.63	0.51	0.28	0.50	0.37
B[a]A	0.24	0.42	0.34	0.25	0.58	0.36	0.26	0.62	0.33	0.22	0.52	0.30	0.67	16.50	3.83	0.27	0.51	0.39	0.51	0.84	0.58	0.27	0.65	0.36
Cry	0.27	0.56	0.45	0.28	0.78	0.48	0.33	0.98	0.44	0.22	0.75	0.36	1.41	22.60	5.74	0.31	0.74	0.53	0.51	1.03	0.64	0.33	0.84	0.48
B[b+k]F	0.39	0.86	0.54	0.38	0.82	0.52	0.39	0.86	0.52	0.35	0.67	0.47	0.83	30.50	6.52	0.40	0.75	0.53	0.78	1.09	0.89	0.41	0.73	0.50
B[a]P	0.28	0.57	0.44	0.32	0.89	0.49	0.29	0.71	0.44	0.26	0.77	0.43	0.55	32.70	6.26	0.31	0.71	0.50	0.57	1.18	0.74	0.34	1.00	0.50
IP	0.47	0.82	0.60	0.46	0.89	0.59	0.44	1.28	0.62	0.41	0.80	0.55	0.59	31.70	6.60	0.45	0.80	0.58	0.90	1.23	1.01	0.47	0.88	0.58
B[g,h,i]P	0.48	0.83	0.64	0.44	0.92	0.60	0.42	1.35	0.61	0.37	0.72	0.51	0.61	30.00	6.42	0.45	0.90	0.65	0.79	1.15	0.94	0.46	0.97	0.62
***Elements* (ng/m^3^) **																								
Fe	5.66	474.00	129.00	5.44	1895.00	397.00	338.00	3815.00	1810.00	13.90	207.00	107.00	1629.00	12982.00	5727.00	87.30	1353.00	497.00	157.00	1700.00	472.00	46.30	168.00	106.00
Mn	2.17	16.80	8.60	5.22	32.30	14.40	3.65	57.60	26.30	0.23	7.54	3.30	33.10	188.00	92.30	3.06	26.30	12.10	4.61	35.50	13.90	1.00	7.00	2.90
Ti	4.88	33.90	12.60	3.40	19.30	9.82	3.49	24.10	11.60	3.31	9.79	6.17	32.00	76.00	48.70	2.41	12.30	7.98	8.34	42.30	19.40	2.10	11.90	5.40
Ni	1.69	8.89	4.96	1.07	48.00	5.05	0.50	48.30	5.80	0.28	2.39	1.14	1.91	18.00	4.73	1.32	10.20	3.74	4.12	14.60	6.64	1.40	10.80	2.90
As	0.08	1.72	0.74	0.23	1.27	0.56	0.12	1.75	0.63	0.09	0.91	0.30	0.59	13.80	4.30	0.10	1.09	0.46	0.13	2.98	1.02	0.10	0.30	0.20
Cd	0.04	0.19	0.10	0.03	0.16	0.09	0.03	0.27	0.11	0.02	0.14	0.06	0.01	0.89	0.29	0.02	0.07	0.04	0.06	0.18	0.12	0.10	0.60	0.10
Zn	6.66	241.39	99.86	5.53	89.77	31.49	7.54	149.27	43.77	2.03	34.39	12.72	20.22	230.35	95.02	1.76	355.45	92.28	25.12	121.33	58.43	16.02	90.39	39.13
Pb	2.88	25.50	8.58	0.52	11.90	4.82	0.50	30.40	7.14	0.66	5.79	2.16	3.51	188.00	53.80	1.31	22.70	6.78	2.79	9.69	5.91	1.50	10.20	4.80
***PM_2.5_* (µg/m^3^) **	11.4	24.2	15.5	6.80	34.5	18.9	7.40	22.5	16.0	5.00	24.9	11.2	14.0	98.5	47.4	7.20	26.5	17.4	9.70	36.0	21.9	6.10	31.0	15.8

* **Fluo**: Fluoranthene, **Anth+Phen**: Anthracene+Phenanthrene, **Pyr**: Pyrene, **B[a]A**: Benzo[a]Anthracene, **Cry**: Crysene, **B[b+k]F**: Benzo[b]Fluoranthene+Benzo[k]Fluoranthene, **B[a]P**: Benzo[a]Pyrene, **IP**: Indeno[1,2,3-cd]Pyrene, **B[g,h,i]P**: Benzo[g,h,i]Perylene.

**Table 4:** PAHs diagnostic ratios.

	B[a]P/B[g,h,i]P	IP/(IP+B[g,h,i]P)	IP/B[g,h,i]P
Traffic	0.5–0.6 [32]	0.17 [33]	0.33 [18,34]
Lead smelter (coke burning)	0.45 [35]	0.36 [35,36]	1.03 [18,34]
Coke combustion	≥1.25 [15]	0.33 [15,36]	
Coal burning	0.9–6.6 [36]	0.56 [33,36]	1.09 [34]
*this study (mean value)*			
*Machiavelli*	0.68	0.49	0.95
*Direzione*	0.82	0.50	1.00
*Parchi*	0.73	0.50	1.02
*Portineria*	0.85	0.52	1.10
*Cokeria*	0.97	0.51	1.00
*Tamburi*	0.76	0.47	0.90
*RIV*	0.78	0.52	1.06
*Adige*	0.82	0.49	0.95

**Table 5:** PAHs and elements airborne concentrations nearby steelworks-related industrial sites worldwide.

Country	Steelworks-Related Industrial Site *	PM_2.5_ Mean Conc. ± SD	B[a]P Mean Conc. ± SD	Elements Mean Conc. ± SD (ng/m^3^) or Min–Max								Ref.
		or Min–Max (µg/m^3^)	or Min–Max (ng/m^3^)	Mn	Fe	Pb	Zn	Cd	As	Ni	Ti	
*Taiwan*	Taichung City ^a,c^	26.6 ± 14.9	0.227 ± 0.166	¯	¯	¯	¯	¯	¯	¯	¯	[37]
	Sha-Lu, Taichung ^e^	39.2 ± 23.6	¯	3.6 ± 2.5	60.9 ± 56.6	8.0 ± 22.0	34.5 ± 16.5	0.2 ± 0.2	8.0 ± 2.4	2.5 ± 2.1	8.4 ± 6.9	[38]
*China*	Hangzhou City ^e^	¯	¯	54.2 ± 5.3	2321.0 ± 788.5	161.6 ± 33.0	729.2 ± 261.2	3.3 ± 0.4	16.8 ± 3.4	5.2 ± 1.4	24.9 ± 2.7	[39]
	Anshan City ^e^	81.22	8.97	¯	¯	¯	¯	¯	¯	¯	¯	[40]
*India*	not specified	¯	0.8 ± 72	¯	¯	¯	¯	¯	¯	¯	¯	[41]
			82.1 ± 129									
	Mangalore ^e^	¯	¯	¯	¯	30 ± 530	300 ± 90	90 ± 40	¯	1820 ± 110	¯	[42]
*United Kingdom*	Port Talbot ^b^	6.5 ± 2.5	¯	3.84 ± 4.8	104 ± 108	4.42 ± 3.88	33.98 ± 86.7	0.26 ± 0.55		0.12 ± 0.17		
		9.2 ± 1.7		12.76 ± 11.8	290 ± 229	8.12 ± 9.27	71.51 ± 117.2	0.88 ± 2.11		0.20 ± 0.33		[43]
*France*	Dunkerque ^b,d^	24.9–33.2	¯	7.19 ± 12.7	92.9 ± 96.1	9.77 ± 8.19	38.7 ± 46.5	0.30 ± 0.63	0.77 ± 1.02	3.23 ± 4.93	1.00 ± 1.63	
				8.11 ± 14.6	101 ± 174	13.9 ± 15.6	44.0 ± 88.5	0.35 ± 0.84	0.91 ± 0.68	4.63 ± 3.68	2.22 ± 2.25	[44]
*Germany*	Duisburg ^e^	¯	¯	50–230	2970–17310	50–80	240–350	1.1–1.3	3.1	5.6–11.9	40–70	[45]
*Italy*	Taranto ^a^	11.2–47.4	0.43–6.26	2.90–92.30	106–5727	2.16–53.80	12.72–99.86	0.04–0.29	0.20–4.30	1.14–6.64	5.40–48.70	this paper
*China*	Ambient Air Quality Standards	75 (24-h)	2.5 (24-h)	¯	¯	500	¯	5	6	¯	¯	[46]
		35 (1-year)	1 (1-year)									
*Taiwan*	Air Quality Standards	35 (24-h)	¯	¯	¯	1000	¯	¯	¯	¯	¯	[47]
		15 (1-year)										
*WHO*	WHO Air Quality Guidelines	25 (24-h)	¯	150	¯	500	¯	5	6.6	25	¯	[48]
		10 (1-year)										
*EU LEGISLATION*	EU Air Quality Standards	25 (1-year)	1			500		5	6	20		[49]

* a, b, c, d, e: sampling periods. a: winter; b: spring; c: summer; d: autumn; e: annual.
